# Real-world outcomes and prognostic factors in primary mediastinal B-cell lymphoma: a multicenter study of 157 patients

**DOI:** 10.1007/s00277-025-06644-z

**Published:** 2025-10-11

**Authors:** Selin Küçükyurt, Oguzhan Koca, Esra Terzi Demirsoy, Serkan Akın, Ali Doğan, Deniz Gören, Ahmet Yiğitbaşı, Osman Şahin, Yıldız İpek, Rafiye Çiftçiler, Fahri Şahin, Meral Uluköylü Mengüç, Işıl Erdoğan Özünal, Özge Soyer Kösemehmetoğlu, Yasemin Özgür, Figen Atalay, Hacer Berna Afacan Öztürk, Merve Yüksel, Nesibe Taşer Kanat, Ayşe Uysal, Utku İltar, Abdulkerim Yıldız, Fatma Keklik Karadağ, Mehmet Baysal, Mehmet Can Uğur, Serkan Güven, İbrahim Ethem Pınar, Özgür Mehtap, İbrahim Barışta, Ahmet Muzaffer Demir, Mahmut Yeral, Cem Selim, Güray Saydam, Işık Kaygusuz Atagündüz, Pınar Tığlıoğlu, İmdat Dilek, Mesut Ayer, Ahmet Kürşad Güneş, Meltem Kurt Yüksel, Ali Ünal, Ozan Salim, Nur Soyer, Elif Birtaş Ateşoğlu, Ahmet Emre Eşkazan

**Affiliations:** 1 Cerrahpaşa Faculty of Medicine, Department of Internal Medicine, Division of Hematology, Istanbul University-Cerrahpaşa, Fatih, Istanbul, Türkiye; 2 Cerrahpaşa Faculty of Medicine, Department of Internal Medicine, Istanbul University-Cerrahpaşa, Istanbul, Türkiye; 3 Faculty of Medicine, Department of Internal Medicine, Division of Hematology, Kocaeli University, Kocaeli, Türkiye; 4 Faculty of Medicine, Department of Internal Medicine, Division of Medical Oncology, Hacettepe University, Ankara, Türkiye; 5 Faculty of Medicine, Department of Internal Medicine, Division of Hematology, Van Yüzüncü Yıl University, Van, Türkiye; 6 Faculty of Medicine, Department of Internal Medicine, Division of Hematology, Haliç University, Istanbul, Türkiye; 7 Faculty of Medicine, Department of Internal Medicine, Division of Hematology, Trakya University, Edirne, Türkiye; 8 Faculty of Medicine, Department of Internal Medicine, Division of Hematology, Adana Başkent University, Adana, Türkiye; 9 Department of Hematology, Kartal Dr. Lütfi Kırdar City Hospital, Istanbul, Türkiye; 10 Faculty of Medicine, Department of Internal Medicine, Division of Hematology, Selçuk University, Konya, Türkiye; 11 Faculty of Medicine, Department of Internal Medicine, Division of Hematology, Ege University, Izmir, Türkiye; 12 Department of Hematology, Marmara University Faculty of Medicine Pendik Training and Research Hospital, Istanbul, Türkiye; 13 Department of Hematology, Göztepe Prof. Dr. Süleyman Yalçın City Hospital, Istanbul, Türkiye; 14 Department of Hematology, Ankara Bilkent City Hospital, Ankara, Türkiye; 15 Department of Hematology, Başakşehir Çam and Sakura City Hospital, Istanbul, Türkiye; 16 Faculty of Medicine, Department of Internal Medicine, Division of Hematology, Yeditepe University, Istanbul, Türkiye; 17 Department of Hematology, Ankara Etlik City Hospital, Ankara, Türkiye; 18 Faculty of Medicine, Department of Internal Medicine, Division of Hematology, Ankara University, Ankara, Türkiye; 19 Faculty of Medicine, Department of Internal Medicine, Division of Hematology, Erciyes University, Kayseri, Türkiye; 20 Faculty of Medicine, Department of Internal Medicine, Division of Hematology, Fırat University, Elazığ, Türkiye; 21 Faculty of Medicine, Department of Internal Medicine, Division of Hematology, Akdeniz University, Antalya, Türkiye; 22 Faculty of Medicine, Department of Internal Medicine, Division of Hematology, Hitit University, Çorum, Türkiye; 23 Department of Hematology, İzmir City Hospital, İzmir, Türkiye; 24 Department of Hematology, Bursa Ali Osman Sönmez Oncology Hospital, Bursa, Türkiye; 25 Department of Hematology, Health Science University, Tepecik Training and Research Hospital, İzmir, Türkiye; 26 Department of Hematology, Çanakkale Mehmet Akif Ersoy State Hospital, Çanakkale, Türkiye; 27 Department of Hematology, Isparta City Hospital, Isparta, Türkiye

**Keywords:** AaIPI, DA-EPOCH-R, IPI, NCCN-IPI, Primary mediastinal B-cell lymphoma, PMBCL, R-CHOP, Radiotherapy

## Abstract

Primary mediastinal B-cell lymphoma (PMBCL) is a rare and distinct subtype of non-Hodgkin lymphoma. No consensus exists on optimal frontline treatment, and the use of R-CHOP ± radiotherapy (RT) and DA-EPOCH-R ± RT remains common, yet comparative real-world data are limited. In our multicenter retrospective study, we analyzed PMBCL patients, stratified by the first-line therapy (R-CHOP-21 ± RT or DA-EPOCH-R ± RT). Primary outcomes were complete response (CR) rate, progression-free survival (PFS), and overall survival (OS), alongside assessment of treatment-related toxicities and prognostic factors for PFS and OS. We included 157 patients [R-CHOP ± RT group (*n* = 80) and DA-EPOCH-R ± RT group (*n* = 77)] with a median age of 31 years, of whom 68.2% were female. CR rates were similar for R-CHOP ± RT (75%) and DA-EPOCH-R ± RT (76.6%). RT use was higher in the R-CHOP group (41.2% vs. 19.5%, *p* = 0.002). DA-EPOCH-R had significantly higher toxicity (29.9% vs. 16.2%, *p* = 0.033). The median follow-up of the entire cohort was 29 months with 2-year PFS and OS rates of 73.9% and 83.6%, respectively. Also, PFS and OS did not differ between regimens. In patients achieving CR with R-CHOP, RT omission did not impact survival. Multivariate analysis identified older age, poor performance status, superior vena cava syndrome and splenic involvement as independent OS predictors, while pericardial effusion, splenic involvement and hemoglobin < 10.5 g/dL were linked to inferior PFS. R-CHOP-21 ± RT and DA-EPOCH-R ± RT provide comparable efficacy in PMBCL. Due to the higher toxicity of DA-EPOCH-R, for those achieving CR following R-CHOP, selective RT omission may be a reasonable alternative. Established and disease-specific prognostic factors should guide individualized treatment strategies.

**Supplementary Information:**

The online version contains supplementary material available at 10.1007/s00277-025-06644-z.

## Introduction

Primary mediastinal B-cell lymphoma (PMBCL) is a rare subtype of non-Hodgkin lymphoma derived from thymic B cells, accounting for approximately 2–4% of all cases [1, 2]. Due to its distinct clinicopathological features, it is recognized by the World Health Organization (WHO) as a unique disease entity [3]. PMBCL typically presents in women during the third or fourth decade of life, often as a bulky mediastinal mass at an early disease stage [4].

To date, no PMBCL-specific prognostic scoring system has been established. The International Prognostic Index (IPI) and age-adjusted IPI (aaIPI) are commonly employed for risk stratification, despite their limitations in this context [5–7].

There are no randomized controlled trials that definitively determine the optimal first-line treatment for PMBCL. Current treatment strategies are largely based on non-randomized prospective and retrospective studies. Recommended regimens include R-CHOP (rituximab, cyclophosphamide, doxorubicin, vincristine, and prednisone) with or without radiotherapy (RT), or dose-adjusted (DA)-EPOCH-R (etoposide, prednisone, vincristine, cyclophosphamide, doxorubicin, and rituximab) alone [4, 8]. The application of RT is particularly challenging in young patients due to long-term risks of cardiopulmonary toxicity and secondary malignancies. Conversely, DA-EPOCH-R is associated with higher toxicity, including febrile neutropenia, infectious complications, and treatment-related hospitalizations [4, 8].

Existing data on treatment response, progression-free survival (PFS), overall survival (OS), and the requirement for radiotherapy (RT) remain inconsistent across studies [9]. Thus, real-life evidence is crucial for better defining outcomes and guiding individualized treatment approaches in PMBCL.

Our objective is to compare the treatment outcomes, therapy-related toxicities, PFS, and OS of R-CHOP-21 ± RT and DA-EPOCH-R ± RT. Additionally, we aim to evaluate the applicability of existing risk scoring systems, as well as identify other potential prognostic factors in PMBCL.

##  Materials and methods

###  Study population

This is a retrospective, multi-center, observational study. All patients aged > 16 years who were newly diagnosed with PMBCL according to the WHO 2008 classification [10] and received at least one cycle of chemoimmunotherapy between 2006 and 2024 were retrospectively identified from 25 hematology centers across Türkiye.

Patients who received treatment before 2006 were excluded from the study, as rituximab was not yet available in Turkey at that time and thus could not be incorporated into standard chemotherapy regimens. Given the retrospective, multicenter design, no a priori sample-size calculation was performed; instead, all eligible patients identified through retrospective chart review were included in the study.

Demographic and clinical characteristics, comorbidities, laboratory assessments, radiological and pathological findings, treatment regimens, treatment responses, treatment-related toxicities (if any) and prognostic assessments were recorded by each participating center. Patients were subsequently grouped according to their first-line treatment, and these variables were compared across the treatment groups.

The study protocol received approval from the İstanbul University-Cerrahpaşa Ethics Committee and was conducted in accordance with the principles of the Declaration of Helsinki.

The primary endpoints of the study were first-line treatment response, PFS and OS. Secondary endpoints encompassed the identification of prognostic clinical and laboratory variables, the assessment of treatment-related toxicity and relapse, and the comparison of treatment regimens with respect to PFS and OS across different risk profiles.

### Treatment

Patients diagnosed with PMBCL who received treatment with R-CHOP-21 ± RT or DA-EPOCH-R ± RT were included in the study. The R-CHOP protocol consisted of prednisolone 100 mg on days 1 to 5, rituximab 375 mg/m^2^ on day 1, cyclophosphamide 750 mg/m^2^ on day 1, doxorubicin 50 mg/m^2^ on day 1, and vincristine 1.4 mg/m^2^ (maximum dose 2 mg) on day 1, administered every 21 days. The DA-EPOCH-R regimen was administered as described in the original publication [11]. Toxicities were graded using the U.S. National Cancer Institute's Common Terminology Criteria for Adverse Events (CTCAE) [12]. RT was delivered at the discretion of the treating physician.

### Evaluation of response

Interim response evaluations were performed after 3 to 4 cycles of first-line therapy, while end-of-treatment assessments were conducted 6 to 8 weeks following the completion of the last treatment cycle. Imaging was performed using either PET/CT or contrast-enhanced CT, depending on availability and clinical context. Treatment response was categorized according to the Lugano classification criteria. Similarly, response assessments by PET-CT scans were performed 12 weeks following the completion of RT [13].

For patients assessed with PET/CT, metabolic response was defined using the Deauville 5-point scale: scores of 1–3 were considered negative, while scores of 4–5 were considered positive. Complete remission (CR) required disappearance of all metabolic activity related to lymphoma, whereas partial remission (PR) was defined as a ≥ 50% reduction in metabolic activity without new lesions.

For patients assessed with contrast-enhanced CT, radiologic response was determined by size-based Lugano criteria. CR was defined as the complete disappearance of all target lesions and lymph nodes < 1.5 cm in the long axis; PR was defined as a ≥ 50% decrease in the sum of the product of diameters of up to six target lesions. Stable disease (SD) and progressive disease (PD) were categorized according to the Lugano definitions.

### Statistical analysis

All data were analyzed by using IBM SPSS Statistics 25.0. Nominal variables were presented as frequencies and percentages. Categorical variables were compared using Pearson’s chi-square test, Fisher’s exact test or Fisher-Freeman-Halton exact test as appropriate. For variables not normally distributed, median with variables range (minimum to maximum) was used. Mann–Whitney U test was used for continuous variables that showed non-normal distribution to test whether there were any differences between the two groups. All tests were two-sided, and *p* < 0.05 was considered statistically significant.

Survival curves were generated using the Kaplan–Meier method. PFS was defined as the time from the date of diagnosis to the date of documented disease progression, relapse, or death from any cause, whichever occurred first. OS was determined by calculating the time between the date of PMBCL diagnosis and the date of death from any cause or the date of the last follow-up (for patients who were still alive). The duration of follow-up was also documented.

Univariate analysis revealed potential prognostic factors associated with PFS and OS. Variables with a significance threshold of *p* < 0.1 in the univariate analysis were incorporated into a multivariate Cox proportional hazards model, and selection was performed using a stepwise approach to assess their impact on survival outcomes, expressed as hazard ratios (HR) with corresponding 95% confidence intervals (CI).

## Results

A total of 157 patients with PMBCL were included, with female predominance (68.2%). The median age at diagnosis was 31 years (range, 17–85 years), and 73.2% of patients presented with early-stage disease. Bulky disease (tumor size ≥ 10 cm) was observed in 54.1% of cases, and B symptoms were present in 46.5% (Table 1).

**Table 1:** Demographic features and characteristics of the patients

Characteristics	Entire cohort (*N* = 157)	R-CHOP-21 (*N* = 80)	DA-EPOCH-R (*N* = 77)	*p* value
Sex, *n* (%)				
Female	107 (68.2)	58 (72.5)	49 (63.6)	0.154
Male	50 (31.8)	22 (27.5)	28 (36.4)	
Age at diagnosis, years				
Median (Range)	31 (17–85)	31.5 (18–80)	31 (17–85)	0.305
Age categories, *n* (%)				
< 60 years	147 (93.6)	72 (90)	75 (97.4)	0.056
≥ 60 years	10 (6.4)	8 (10)	2 (2.6)	
SVCS, n (%)	43 (27.4)	19 (23.7)	24 (31.2)	0.194
Pericardial effusion, *n* (%)	60 (38.2)	30 (37.5)	30 (39.0)	0.49
Pleural effusion, *n* (%)	75 (47.8)	37 (46.2)	38 (49.3)	0.409
B symptoms, *n* (%)	73 (46.5)	35 (43.7)	38 (49.3)	0.294
ECOG, *n* (%)				
0–1	143 (91.1)	71 (88.7)	72 (93.5)	0.223
≥ 2	14 (8.9)	9 (11.3)	5 (6.5)	
Tumor size at diagnosis, *n* (%)				
≥ 10 cm	85 (54.1)	39 (48.7)	46 (59.7)	0.188
< 10 cm	68 (43.3)	37 (46.3)	31 (40.3)	
N/A	4 (2.6)	4 (5)	-	
Median SUVmax of the mass at diagnosis, *n* (%)				
< 20	58 (36.9)	33 (41.2)	25 (32.5)	0.080
≥ 20	70 (44.6)	30 (37.5)	40 (51.9)	
N/A	29 (18.5)	17 (21.3)	12 (15.6)	
Ki67 proliferation index				
≤ 50	12 (7.6)	6 (7.5)	6 (7.8)	0.492
> 50	115 (73.3)	52 (65)	63 (81.8)	
N/A	30 (19.1)	22 (27.5)	8 (10.4)	
CD30 expression, *n* (%)				
Positive	119 (75.8)	60 (75)	59 (76.6)	0.371
Negative	21 (13.4)	12 (15)	9 (11.7)	
N/A	17 (10.8)	8 (10)	9 (11.7)	
Extranodal involvement, excluding the primary mass				
Yes	23 (14.6)	11 (13.7)	12(15.6)	0.46
Bone marrow involvement, *n* (%)				
Yes	6 (3.8)	3 (3.7)	3 (3.9)	0.641
Liver involvement, *n* (%)				
Yes	4 (2.5)	1 (1.2)	3 (3.9)	0.296
Splenic involvement, *n* (%)				
Yes	6 (3.8)	3 (3.7)	3 (3.9)	0.641
Infradiaphragmatic LN involvement, *n* (%)				
Yes	24 (15.3)	15 (18.7)	9 (11.7)	0.157
Stage, *n* (%)				
Early (I-II)	115 (73.2)	59 (73.7)	56 (72.7)	0.514
Advanced (III-IV)	42 (26.8)	21 (26.3)	21 (27.3)	
IPI, *n* (%)				
0–1	112 (71.3)	58 (72.5)	54 (70.1)	0.44
≥ 2	45 (28.7)	22 (27.5)	23 (29.9)	
NCCN- IPI, *n* (%)				
0–1	70 (44.6)	36 (45)	34 (44.2)	0.522
≥ 2	87 (55.4)	44 (55)	43 (55.8)	
aaIPI, n (%)*				
0–1	114 (72.6)	57 (77)	57 (75)	0.461
2–3	36 (22.9)	17 (23)	19 (25)	
Hemoglobin (g/dL), *n* (%)				
< 10.5	20 (12.7)	9 (11.2)	11 (14.3)	0.37
≥ 10.5	135 (86.0)	70 (87.5)	65 (84.4)	
N/A	2 (1.3)	1 (1.3)	1 (1.3)	

[*aaIPI was calculated exclusively for patients aged ≤ 60 years and the percentages were derived based on this subgroup (*N* = 150)] (*aaIPI* Age-adjusted International Prognostic Index, *DA-EPOCH-R* dose adjusted etoposide-prednisone-vincristine-cyclophosphamide-doxorubicin and rituximab, *ECOG* Eastern Cooperative Oncology Group Performance Status, *IPI* International Prognostic Index, *NCCN-IPI* National Comprehensive Cancer Network – IPI, *R-CHOP-21* rituximab-cyclophosphamide-doxorubicin-vincristine-prednisone, *SUVmax* maximum standardized uptake value, *SVCS* superior vena cava syndrome)

Of the cohort, 51% (*n* = 80) received R-CHOP-21 and 49% (*n* = 77) received DA-EPOCH-R as first-line therapy. Baseline clinical and pathological characteristics, including age, ECOG performance status, tumor size, initial presentation of disease, disease stage, and risk scores (IPI, NCCN-IPI, aaIPI), were comparable between the R-CHOP-21 and DA-EPOCH-R groups (all *p* = NS) (Table 1).

Treatment responses at interim assessment, at the end of first-line immunochemotherapy, and after completion of radiotherapy were shown in Table 2. Overall, 89.2% of patients achieved a PR or better at interim evaluation with no significant difference between treatment groups (*p* = 0.406). At the end of immunochemotherapy, CR rates were 62.5% in the R-CHOP-21 group and 67.5% in the DA-EPOCH-R group; after RT, CR rates increased to 75.0% and 76.6%, respectively. Response rates remained comparable between groups (*p* = 0.267 and *p* = 0.417). Notably, RT utilization was significantly higher among patients treated with R-CHOP-21 than among those receiving DA-EPOCH-R (41.2% vs. 19.5%, *p* = 0.002) (Table 2). When cases in which RT was administered for tumor debulking before systemic therapy were excluded, consolidative RT was significantly more frequently used in patients treated with R-CHOP-21 compared to those who received DA-EPOCH-R (*p* = 0.022) (Table 2).

**Table 2:** Interim and end-of-treatment responses, adverse events and treatment details of the study cohort

Characteristics	Entire cohort (*N* = 157)	R-CHOP-21 (*N* = 80)	DA-EPOCH-R (*N* = 77)	*p* value
Interim response status, *n* (%)				
CR	52 (33.1)	21 (26.3)	31 (40.3)	0.124^**#**^
PR	88 (56.1)	51 (63.8)	37 (48)	
SD	7 (4.5)	4 (5)	3 (3.9)	
PD	1 (0.6)	1 (1.2)	0 (0)	
N/A	9 (5.7)	3 (3.7)	6 (7.8)	
Interim response status, *n* (%)				
≥ PR	140 (89.2)	72 (90)	68 (88.3)	0.406
Response status at the end of chemoimmunotherapy, *n* (%)				
CR	102 (65)	50 (62.5)	52 (67.5)	0.267
Non-CR	52 (33.1)	29 (36.3)	23 (29.9)	
N/A	3 (1.9)	1 (1.3)	2 (2.6)	
RT Exposure, *n* (%)	48 (30.6)	33 (41.2)	15 (19.5)	**0.002**
Reason for RT, *n* (%)*				
Residual mass	26 (54.2)	16 (48.5)	10 (66.7)	**0.022**^**¥**^
Consolidative RT	16 (33.3)	15 (45.4)	1 (6.7)	
Debulking	6 (12.5)	2 (6.1)	4 (26.6)	
Receiving intrathecal therapy for CNS prophylaxis, *n* (%)	22 (13.2)	10 (12.5)	12 (15.6)	0.372
Response status at the end of first-line treatment including RT, *n* (%)				
CR	119 (75.8)	60 (75.0)	59 (76.6)	0.417
Non-CR	34 (21.6)	19 (23.8)	15 (19.5)	
N/A	4 (2.5)	1 (1.3)	3 (3.9)	
Adverse events	36 (22.9)	13 (16.2)	23 (29.9)	**0.033**
Hematologic^§^	25 (69.4)	10 (76.9)	15 (65.2)	
Non-hematologic^§^	11 (30.5)	3 (23.1)	8 (34.8)	

(^#^Fisher-Freeman-Halton exact test, *These numbers represent the subgroup distribution of patients who received radiotherapy, categorized according to the respective clinical indications ^¥^Patients who received radiotherapy for debulking prior to first-line treatment were not included in the comparison between the two groups ^§^Percentages were calculated within the subgroup of patients who experienced adverse events) (*CNS* central nervous system, *CR* complete remission, *DA-EPOCH-R* dose adjusted etoposide- prednisone- vincristine- cyclophosphamide- doxorubicin and rituximab, *PD* progressive disease, *PR* partial remission, *R-CHOP21* rituximab-cyclophosphamide-doxorubicin-vincristine-prednisone, *RD* refractory disease, *RT* radiotherapy, *SD* stable disease)

**Fig. 1:**
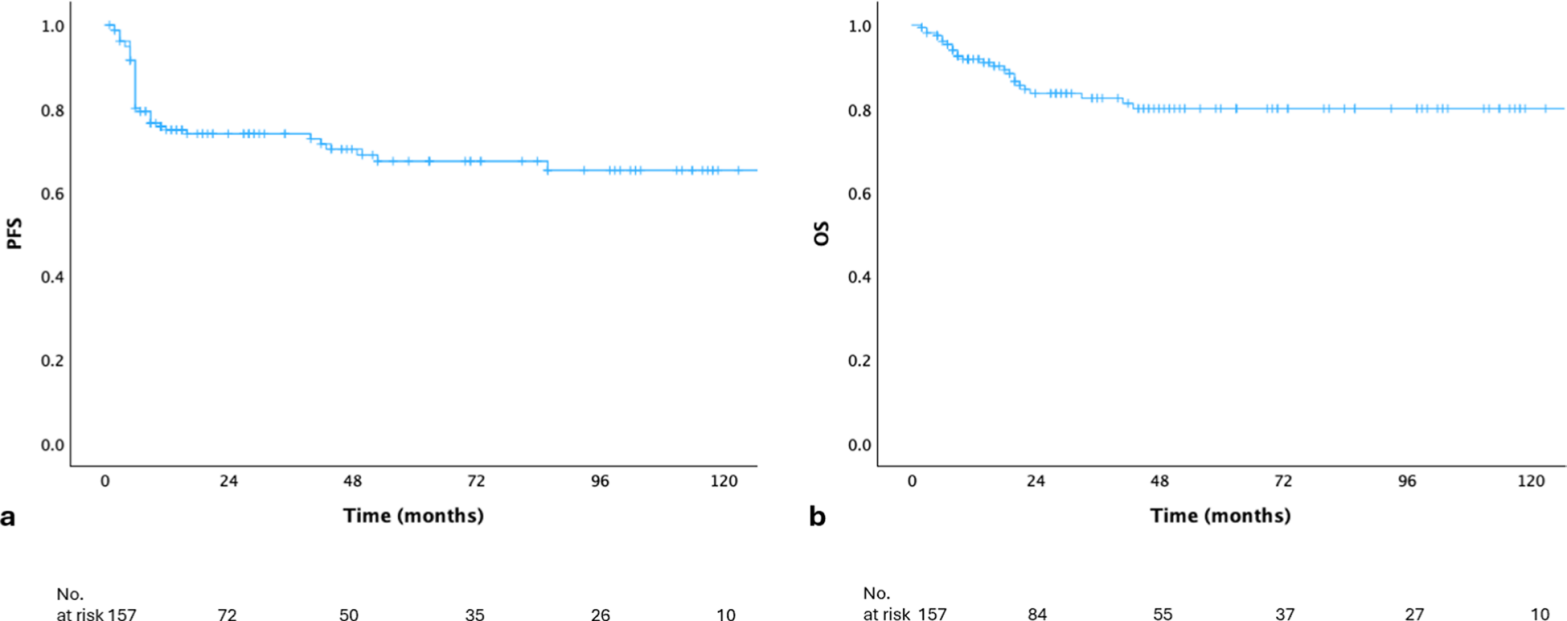
(**a**) Progression-free survival and (**b**) overall survival curves for the entire cohort (OS, overall survival; PFS, progression-free survival)

**Fig. 2:**
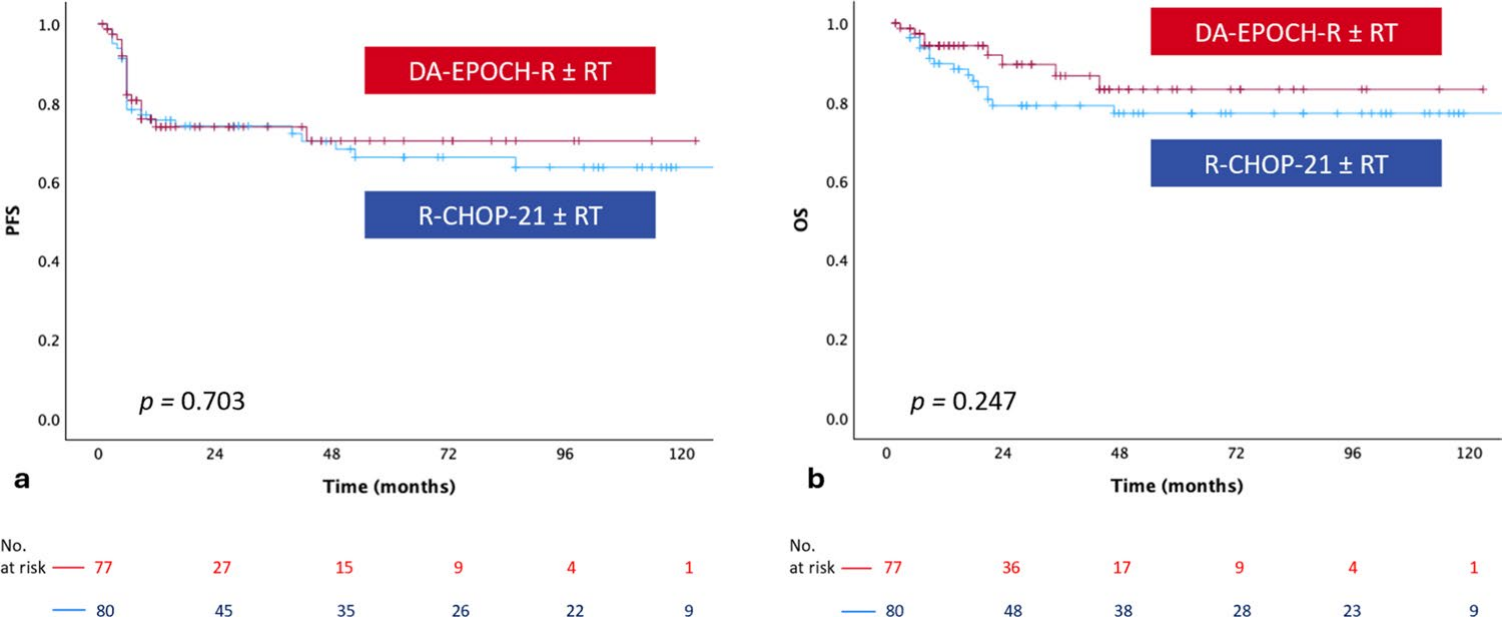
Comparison of DA-EPOCH-R ± RT and R-CHOP21 ± RT groups regarding progression-free survival (**a**) and overall survival (**b**) (DA-EPOCH-R, dose-adjusted etoposide-prednisone-vincristine-cyclophosphamide-doxorubicin and rituximab; OS, overall survival; PFS, progression-free survival; R-CHOP21, rituximab-cyclophosphamide-doxorubicin-vincristine-prednisone; RT, radiotherapy)

The median follow-up duration was 29 months (range, 2–216 months). PFS and OS curves for the entire cohort were presented in Fig. 1a and b. The 2-year PFS and OS rates for the whole cohort were 73.9% and 83.6%. Among patients treated with R-CHOP-21 ± RT and DA-EPOCH-R ± RT, the 2-year PFS rates were 74% and 73.8%, while the corresponding 2-year OS rates were 85.3% and 89.5%, respectively. There was no statistically significant difference in PFS or OS between the two groups (*p* = 0.703 and *p* = 0.247) (Fig. 2a and b).

**Fig. 3:**
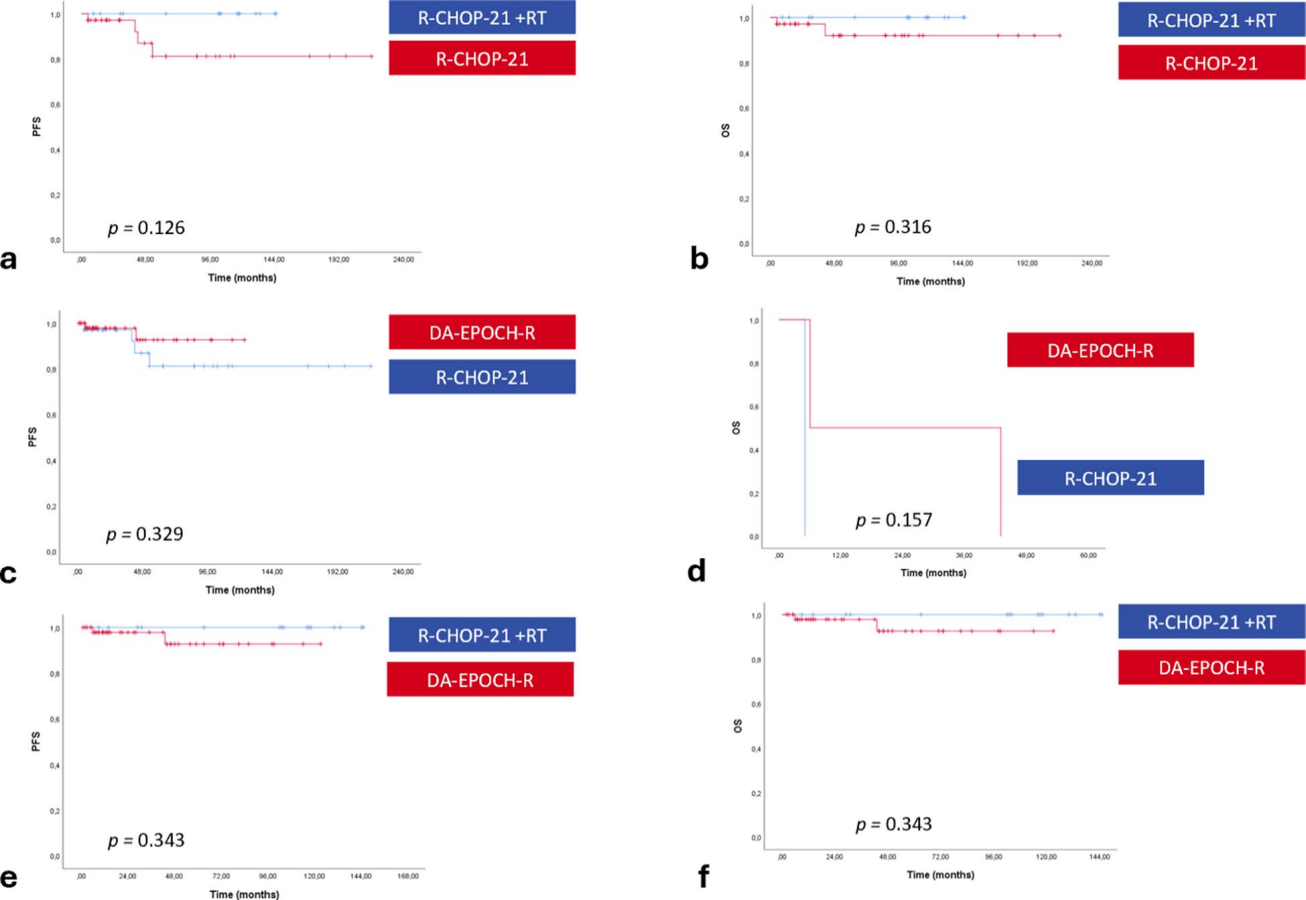
Progression-free survival (panels **a**, **c**, **e**) and overall survival (panels **b**, **d**, **f**) were compared among patients treated with R-CHOP-21 followed by consolidative RT (*N* = 15) versus those without consolidative RT (*N* = 35) (panels a and b, DA-EPOCH-R (*N* = 51) versus R-CHOP-21 without consolidative RT (*N* = 35) (panels c and d), and R-CHOP-21 followed by consolidative RT (*N* = 15) versus DA-EPOCH-R (*N* = 51) (panels **e** and **f**) (DA-EPOCH-R, dose-adjusted etoposide-prednisone-vincristine-cyclophosphamide-doxorubicin and rituximab; OS, overall survival; PFS, progression-free survival; R-CHOP21, rituximab-cyclophosphamide-doxorubicin-vincristine-prednisone; RT, radiotherapy)

Importantly, among patients achieving CR after R-CHOP-21, omission of RT yielded PFS and OS outcomes comparable to those of the DA-EPOCH-R group (*p* = 0.329 and *p* = 0.157) (Fig. 3c and d). Comparisons of PFS and OS between patients who achieved CR after R-CHOP-21 with or without consolidative RT, as well as those receiving consolidative RT following R-CHOP-21 versus DA-EPOCH-R, were displayed in Fig. 3a, b and e, f, respectively. Again, no statistically significant differences were observed between the groups (all *p* = NS) (Fig. 3).

However, DA-EPOCH-R was associated with significantly higher chemotherapy-related toxicity (29.9% vs. 16.2%, *p* = 0.033), predominantly hematologic (65.2%). The most frequent non-hematologic adverse events (AEs) were gastrointestinal toxicities and neurotoxicity. Details of hematologic and non-hematologic AEs for both regimens were provided in Supplementary Table 1.

Primary induction failure was observed in 21.6% of patients (*n* = 34), of whom 12 patients (7.6%) were biopsy-confirmed. The rates of relapsed/refractory disease, use of salvage therapies, and consolidation with autologous stem cell transplantation were similar between groups (all *p* = NS) (Supplementary Table 2).

Univariate analysis demonstrated that multiple clinical parameters—including age, superior vena cava syndrome (SVCS), pericardial effusion, splenic involvement, infradiaphragmatic lymph node involvement, ECOG performance status, Ann Arbor stage, hemoglobin level, and prognostic indices such as IPI, NCCN-IPI, and aaIPI—were significantly associated with survival outcomes (all *p* < 0.05) (Table 3). In multivariate analysis, advanced age, poor ECOG performance status, the presence of SVCS and splenic involvement were independently associated with inferior OS. For PFS, the presence of pericardial effusion, splenic involvement and baseline hemoglobin < 10.5 g/dL emerged as independent predictors of poorer prognosis (Table 3).

**Table 3:** Univariate and multivariate analyses of progression-free and overall survival of the entire cohort (*N* = 157)

		Univariate		Multivariate		Univariate		Multivariate	
Characteristics	Variables	PFS-HR	*p* value	PFS-HR	*p* value	OS-HR	*p* value	OS-HR	*p* value
Median age at diagnosis, years		1.01 (0.99–1.03)	0.237			**1.04 (1.01–1.06)**	**0.005**	**1.03 (1.01–1.06)**	**0.012**
Age at diagnosis, years	< 45								
	≥ 45	1.84 (0.93–3.65)	0.080			**3.28 (1.45–7.44)**	**0.004**		
Sex	Female								
	Male	1.26 (0.69–2.30)	0.456			1.13 (0.50—2.55)	0.773		
SVCS	Yes	1.48 (0.80–2.72)	0.211			1.98 (0.90–4.38)	0.090	**2.78 (1.12–6.94)**	**0.028**
Pericardial effusion	Yes	**1.96 (1.08–3.54)**	**0.026**	**1.99 (1.07–3.70)**	**0.029**	1.81 (0.81–4.05)	0.146		
Pleural effusion	Yes	1.34 (0.74–2.41)	0.335			2.06 (0.90–4.70)	0.085		
B symptoms	Yes	1.35 (0.75–2.42)	0.321			1.01 (0.46–2.23)	0.984		
ECOG	0–1								
	≥ 2	**2.71 (1.25–5.84)**	**0.011**			**6.00 (2.47–14.58)**	** < 0.001**	**4.01 (1.52–10.60)**	**0.005**
Tumor size at diagnosis	< 10 cm								
	≥ 10 cm	1.70 (0.88–3.27)	0.112			2.23 (0.88–5.61)	0.089		
Median SUVmax of the mass at diagnosis		1.01 (0.97–1.05)	0.607			0.97 (0.91—1.04)	0.389		
Bone marrow involvement	Yes	2.22 (0.69–7.17)	0.183			1.34 (0.18–9.94)	0.773		
Splenic involvement	Yes	**3.51 (1.25–9.84)**	**0.017**	**5.20 (1.78–15.23)**	**0.003**	**5.52 (1.64–18.61)**	**0.006**	**9.81 (2.52–38.11)**	** < 0.001**
Infradiaphragmatic LN involvement	Yes	**2.69 (1.40–5.14)**	**0.003**			2.34 (0.97–5.64)	0.059		
Ann Arbor stage	1–2								
	3–4	**2.72 (1.50–4.92)**	** < 0.001**			**2.46 (1.10–5.49)**	**0.028**		
Ki67 proliferation index	≤ 50	1.09 (0.39–3.06)	0.875			1.68 (0.49–5.75)	0.406		
	> 50								
IPI	0–1								
	≥ 2	**2.28 (1.26–4.14)**	**0.007**			**2.85 (1.30–6.25)**	**0.009**		
NCCN-IPI	0–1								
	≥ 2	**2.38 (1.24–4.54)**	**0.009**			**2.56 (1.06–6.20)**	**0.037**		
aaIPI*	0–1								
	2–3	**2.36 (1.24–4.48)**	**0.009**			**2.75 (1.16–6.53)**	**0.022**		
Hemoglobin (g/dL)	< 10.5	**3.05 (1.57–5.92)**	**0.001**	**2.96 (1.50–5.83)**	**0.002**	2.14 (0.85–5.39)	0.107		

(*aaIPI was calculated exclusively for patients aged ≤ 60 years (*N* = 150)) (*aaIPI* Age-adjusted International Prognostic Index, *ECOG* Eastern Cooperative Oncology Group Performance Status, *HR* hazard ratio, *IPI* International Prognostic Index, *LN* lymph node, *NCCN-IPI* National Comprehensive Cancer Network – IPI, *OS* overall survival, *PFS* progression-free survival, *SUVmax* maximum standardized uptake value)

The effect of R-CHOP-21 ± RT and DA-EPOCH-R ± RT regimens on PFS and OS was evaluated across subgroups potentially associated with increased risk (Table 4). Among patients with an IPI, NCCN-IPI, or aaIPI score of 2 or higher, PFS and OS outcomes did not differ significantly between the treatment regimens. No significant differences in PFS or OS were observed between the two regimens in the presence of B symptoms, bulky disease, pericardial effusion, or SVCS. Similarly, advanced-stage disease, involvement of splenic and infradiaphragmatic lymph nodes, and hemoglobin levels < 10.5 g/dL were associated with comparable PFS and OS outcomes between the treatment arms. Notably, among patients with pleural effusion, those treated with DA-EPOCH-R ± RT had significantly better PFS and OS (*p* = 0.042 and *p* = 0.046, respectively) (Table 4).

**Table 4:** Effect of DA-EPOCH-R ± RT versus R-CHOP-21 ± RT on PFS and OS across clinically high-risk subgroups: Univariate Analysis

	Univariate analysis	
Variables	PFS - HR	OS - HR
IPI ≥ 2	0.73 (0.29–1.81)* p* = 0.491	0.31 (0.09–1.16) *p* = 0.083
NCCN-IPI ≥ 2	0.81 (0.40–1.66) *p* = 0.571	0.57 (0.21–1.51) *p* = 0.257
aaIPI ≥ 2*	0.82 (0.30–2.27) *p* = 0.704	0.24 (0.05–1.18) *p* = 0.079
B symptoms	0.63 (0.28–1.46) *p* = 0.281	0.34 (0.09–1.24) *p* = 0.103
Presence of bulky disease	0.75 (0.36–1.56) *p* = 0.437	0.62 (0.24–1.60) *p* = 0.322
Pericardial effusion	0.64 (0.27–1.47) *p* = 0.290	0.46 (0.14–1.50) *p* = 0.198
Pleural effusion	0.40 (0.17–0.97) ***p* = 0.042**	0.32 (0.10–0.98) ***p* = 0.046**
SVCS	0.75 (0.27–2.10) *p* = 0.579	0.52 (0.15–1.84) *p* = 0.312
Advanced-stage disease	0.64 (0.25–1.60) *p* = 0.339	0.25 (0.05–1.14) *p* = 0.072
Splenic involvement	0.37 (0.04–3.54) *p* = 0.385	0.01 (0–141.3) *p* = 0.351
Infradiafragmatic LN involvement	0.54 (0.15–1.98) *p* = 0.352	0.22 (0.03–1.86) *p* = 0.165
Hemoglobin < 10.5 g/dL	0.72 (0.23–2.29) *p* = 0.580	0.46 (0.08–2.53) *p* = 0.373

(*aaIPI was calculated exclusively for patients aged ≤ 60 years) (*aaIPI* Age-adjusted International Prognostic Index, *HR* hazard ratio, *IPI* International Prognostic Index, *LN* lymph node, *NCCN-IPI* National Comprehensive Cancer Network – IPI, *OS* overall survival, *PFS* progression-free survival, *SVCS* superior vena cava syndrome)

## Discussion

Previous studies have demonstrated a clear survival advantage with rituximab-containing protocols compared to rituximab-free regimens in the treatment of PMBCL. However, to date, in the rituximab era, a consensus has yet to be reached on the optimal frontline therapy for PMBCL due to the lack of randomized controlled studies. Our retrospective study, comprising one of the largest real-world cohorts, provides valuable comparative data on the efficacy and toxicity profiles of two frequently used regimens, while also identifying prognostic factors that may inform long-term outcomes. As expected, our cohort predominantly consisted of young female patients presenting with bulky mediastinal masses, consistent with the typical clinical phenotype of PMBCL [1, 2, 4].

One of the major findings of our study is the comparable efficacy of R-CHOP-21 ± RT and DA-EPOCH-R ± RT regimens. CR rates were almost identical between the two groups (75% vs. 76.6%), and there were no statistically significant differences in PFS or OS. Importantly, in two previously published studies with among largest reported cohorts of PMBCL patients treated with DA-EPOCH-R ± RT and R-CHOP ± RT, survival outcomes were broadly consistent with those observed in our study, but PFS and OS were slightly lower in both arms in our cohort [14, 15].

Consistent with our findings, similar survival rates have been reported between the DA-EPOCH-R and R-CHOP groups in most cohorts. Although response rates varied, RT was used significantly less frequently in the DA-EPOCH-R group compared to the R-CHOP group, and treatment-related toxicity was more common in the DA-EPOCH-R group [16]. In our cohort, the more frequent use of RT in the R-CHOP group (41.2%) raises important concerns about long-term toxicity, particularly in young adults. As previously emphasized in the literature [17], these concerns primarily relate to potential risks such as late-onset cardiopulmonary complications and secondary malignancies in this population [16–18].

The role of RT in PMBCL remains a matter of ongoing debate. Importantly, in our cohort, the omission of RT in patients who achieved CR following R-CHOP did not negatively impact PFS or OS when compared to those who were treated with DA-EPOCH-R. This finding highlights the potential for safely reducing RT in carefully selected patients. Similar observations have been reported, indicating that long-term survival was not compromised when RT was omitted after immunochemotherapy in patients who achieved PET negativity [19, 20].

Notably, our data demonstrated that neither bulky disease, high IPI, nor advanced stage significantly affected the comparative outcomes between treatment groups (Table 4). These results contrast somewhat with prior concerns that R-CHOP may be suboptimal in high-risk patients. For instance, Chan et al. [17] reported inferior 5-year PFS in patients receiving R-CHOP alone compared to those treated with R-CHOP + RT or DA-EPOCH-R, particularly among patients with bulky disease [17]. Nevertheless, our findings support the notion that R-CHOP ± RT remains a viable and effective treatment option in real-world settings, including in patients with bulky or advanced-stage disease, when appropriately selected and managed.

Toxicity profiles varied significantly between treatment regimens, with DA-EPOCH-R associated with a higher rate of AEs (29.9%), predominantly hematologic, consistent with prior studies [16]. As hematological toxicity (such as febrile neutropenia) frequently occurred (AEs), vigorous primary prophylaxis against infections, with long-acting G-CSF, trimethoprim- sulfamethoxazole and acyclovir should be recommended in PMBCL patients receiving anthracycline-based regimens [21]. These results highlight the importance of tailoring treatment intensity based on individual patient characteristics and institutional resources—particularly in resource-constrained settings where the feasibility of intensive regimens may be limited by access to hospitalization and supportive care. On the other hand, recent advances suggest that alternative anthracycline formulations may mitigate toxicity while preserving efficacy. In particular, non-pegylated liposomal doxorubicin (NPLD)–containing regimens have shown promising results. Picardi and colleagues [22] reported that, in newly diagnosed patients with PMBCL, a dose-intensified NPLD-based first-line regimen (R-COMP-DIX6) resulted in a high complete metabolic remission rate of 89% with a favorable tolerability and safety profile. Incorporating such approaches into future treatment strategies may offer an effective means of reducing anthracycline-associated morbidity without compromising disease control.

In our cohort, established prognostic indices including the IPI, NCCN-IPI, and aaIPI demonstrated significant predictive value for both PFS and OS in patients with PMBCL. In the multivariate analysis, advanced age, impaired ECOG status, SVCS, and splenic involvement were identified as independent predictors of worse OS. Regarding PFS, advanced disease stage at diagnosis, presence of pericardial effusion, splenic involvement and baseline hemoglobin level below 10.5 g/dL were independently linked to inferior prognosis. These results partially diverge from earlier reports. For example, in the study by Hamlin et al. [7], the aaIPI failed to demonstrate prognostic utility in PMBCL, possibly reflecting limitations of traditional indices in capturing disease-specific biology [7]. In contrast, a recent study identified several clinical features, including age, receipt of chemotherapy, and Ann Arbor stage, as prognostically relevant in PMBCL—findings that are broadly consistent with ours. Collectively, our results support the relevance of both classic and PMBCL-specific clinical parameters in prognostication and highlight the need for tailored risk-stratification tools in this unique lymphoma subtype [23].

In our study, the primary refractory disease rate was high at 21.6% (Supplementary Table 2). In PMBCL, particularly in cases presenting with large tumor masses or lesions with a substantial fibrotic component at diagnosis, residual mediastinal masses frequently persist at the end of treatment [4, 8, 24]. This poses an ongoing challenge in the interpretation of post-treatment PET imaging. Notably, our cohort included patients whose treatment response was assessed using CT alone. In our analysis, the presence of post-treatment mediastinal masses was not a reliable predictor of active disease, consistent with prior reports showing a low positive predictive value of 42% for end-of-treatment PET scans with Deauville scores of 4–5 [14]. These findings likely reflect post-treatment inflammatory or fibrotic changes rather than residual lymphoma and support the NCCN recommendation to confirm suspected residual disease by biopsy in non-responding patients [25]. Importantly, patients with Deauville score 5 at end-of-treatment PET had significantly shorter time to progression, highlighting the value of incorporating Deauville scoring into risk-adapted follow-up strategies.

Nonetheless, our study has certain limitations. Its retrospective design may introduce selection bias, particularly in treatment allocation and radiotherapy decisions, which were made at the discretion of treating physicians. Additionally, the lack of standardization in PET/CT imaging across centers may have affected the consistency of response assessments. Also, the retrospective and multicenter nature of this study likely contributed to the lower-than-expected incidence of particularly non-hematologic AEs, which should be acknowledged as one of its limitations. Given the median follow-up of 29 months, the study may be underpowered to detect late AEs—particularly secondary malignancies and radiation-associated cardiotoxicity—and conclusions regarding long-term safety should therefore be interpreted with caution. Despite these limitations, the study's large sample size and nationwide, multicenter representation enhance the generalizability of our findings. Moreover, the identification of clinically relevant prognostic factors contributes meaningfully to risk stratification and may inform individualized treatment approaches in PMBCL.

## Conclusion

In this large real-life cohort of PMBCL patients, R-CHOP-21 ± RT and DA-EPOCH-R ± RT demonstrated comparable efficacy in terms of CR rates, PFS, and OS, although DA-EPOCH-R was associated with higher toxicity. Our findings suggest that RT may be safely omitted in selected patients achieving CR with R-CHOP-21. Furthermore, the IPI, NCCN-IPI, and aaIPI can be reliably applied to predict prognosis in PMBCL. Notably, additional clinical parameters such as baseline hemoglobin level, splenic involvement, SVCS, and pericardial effusion at diagnosis may serve as valuable prognostic indicators and merit consideration for inclusion in future PMBCL-specific risk models. Prospective validation of these findings is warranted to refine individualized treatment strategies in this distinct lymphoma subtype.

## Supplementary Information

Below is the link to the electronic supplementary material.

Supplementary file1 (DOCX 16 KB)

Supplementary file2 (DOCX 23 KB)

## Data Availability

The datasets used and/or analysed during the current study are available from the corresponding author on reasonable request.

## Declarations

### Permission to reproduce material from other sources

No previously published material was reproduced in this manuscript.

### Clinical trial registration (including trial number)

Patients were not included in any clinical trial.
