# Supplementary material for: Real-world outcomes and prognostic factors in primary mediastinal B-cell lymphoma: a multicenter study of 157 patients
Source: Ann Hematol. 2025 Oct 11;104(9):4679–90. doi: 10.1007/s00277-025-06644-z (PMC12552371; doi:10.1007/s00277-025-06644-z)
Supplement: Supplementary file 1 — Supplementary file1 (DOCX 16 KB) [file 277_2025_6644_MOESM1_ESM.docx]

**Supplementary Table 1.** Grade 3-4 hematologic and non-hematologic adverse events by treatment regimen (R-CHOP-21 vs DA-EPOCH-R).

|  | **R-CHOP-21**  **(N = 11)** | **DA-EPOCH-R**  **(N = 15)** |
| --- | --- | --- |
| **Hematologic, n (%)** | **n = 10 (90.9)** | **n = 15 (100)** |
| Anemia, n | 1 | 4 |
| Neutropenia, n | 10 | 15 |
| Neutropenic fever, n | 2 | 8 |
| Thrombocytopenia, n | 1 | 2 |
| **Non-hematologic, n (%)** | **n = 1 (9.1)** | **n = 0** |
| Hyponatremia, n | 1 | 0 |
