# Supplementary material for: Real-world outcomes and prognostic factors in primary mediastinal B-cell lymphoma: a multicenter study of 157 patients
Source: Ann Hematol. 2025 Oct 11;104(9):4679–90. doi: 10.1007/s00277-025-06644-z (PMC12552371; doi:10.1007/s00277-025-06644-z)
Supplement: Supplementary file 2 — Supplementary file2 (DOCX 23 KB) [file 277_2025_6644_MOESM2_ESM.docx]

| **Characteristics** | **Entire Cohort**  **(N = 39)** | **R-CHOP-21**  **(N = 23)** | **DA-EPOCH-R**  **(N = 16)** | ***p* value** |
| --- | --- | --- | --- | --- |
| Disease Status, n (%)  Relapsed  Refractory | 5 (3.2)  34 (21.6) | 4 (5)  19 (23.7) | 1 (1.3)  15 (19.5) | 0.187  0.325 |
| Time of Relapse, n (%)  <24 mo >24 mo | 0  5 (100) | 0  4 (100) | 0  1 (100) | 0.187 |
| Lines of salvage therapies, n (%) 1 2 >2  N/A | 17 (43.6)  7 (17.9)  8 (20.5)  7 (17.9) | 7 (30.4)  5 (21.7)  6 (26.0)  5 (21.7) | 10 (62.5)  2 (12.5)  2 (12.5)  2 (12.5) | 0.226^#^ |
| Autologous stem cell transplantation, n (%) | 23 (58.9) | 11 (47.8) | 12 (75) | 0.364 |
| BV maintenance following autologous transplantation, n (%) | 2 (5.1) | 1 (9) | 1 (8.3) | -^*^ |
| Allogeneic stem cell transplantation, n (%) | 2 (5.1) | 1 (4.3) | 1 (6.3) | -^*^ |

**Supplementary Table 2.** Post-treatment disease course and salvage strategies in relapsed or refractory disease (#Fisher-Freeman-Halton exact test *Due to the limited number of patients in these subgroups, no statistical comparison was performed between treatment groups (BV, Brentuximab vedotin; DA-EPOCH-R, dose adjusted etoposide-prednisone- vincristine-cyclophosphamide-doxorubicin and rituximab; R-CHOP-21, rituximab-cyclophosphamide-doxorubicin-vincristine-prednisone; mo, months).
